# Melatonin Inhibits Glucose-Induced Apoptosis in Osteoblastic Cell Line Through PERK-eIF2α-ATF4 Pathway

**DOI:** 10.3389/fphar.2020.602307

**Published:** 2020-12-16

**Authors:** Renyi Zhou, Yue Ma, Zhengbo Tao, Shui Qiu, Zunlei Gong, Lin Tao, Yue Zhu

**Affiliations:** ^1^ Department of Orthopedics, The First Hospital of China Medical University, Shenyang, China; ^2^ Department of Pulmonary and Critical Care Medicine, Shengjing Hospital of China Medical University, Shenyang, China

**Keywords:** melatonin, osteoblast, endoplasmic reticulum stress, apoptosis, PERK-eIF2a-ATF4

## Abstract

Osteoporosis is a common disease resulting in deteriorated microarchitecture and decreased bone mass. In type 2 diabetes patients, the incidence of osteoporosis is significantly higher accompanied by increased apoptosis of osteoblasts. In this study, using the osteoblastic cell line MC3T3-E1, we show that high glucose reduces cell viability and induces apoptosis. Also, high glucose leads to endoplasmic reticulum (ER) stress (ERS) via an increase in calcium flux and upregulation of the ER chaperone binding immunoglobulin protein (BiP). Moreover, it induces post-translational activation of eukaryotic initiation factor 2 alpha (eIF2α) which functions downstream of PKR-like ER kinase (PERK). This subsequently leads to post-translational activation of the transcription factor 4 (ATF4) and upregulation of C/EBP-homologous protein (CHOP) which is an ER stress-induced regulator of apoptosis, as well as downstream effectors DNAJC3, HYOU1, and CALR. Interestingly, melatonin treatment significantly alleviates the high-glucose induced changes in cell growth, apoptosis, and calcium influx by inhibiting the PERK-eIF2α-ATF4-CHOP signaling pathway. Additionally, the MC3T3-E1 cells engineered to express a phosphodead eIF2α mutant did not show high glucose induced ER stress, confirming that melatonin protects osteoblasts against high-glucose induced changes by decreasing ER-stress induced apoptosis by impacting the PERK-eIF2α-ATF4-CHOP signaling pathway. The protective of melatonin against high glucose-induced ER stress and apoptosis was attenuated when the cells were pre-treated with a melatonin receptor antagonist, indicating that the effect of melatonin was mediated via the melatonin receptors in this context. These findings lay the provide mechanistic insights of melatonin’s protective action on osteoblasts and will be potentially be useful in ongoing pre-clinical and clinical studies to evaluate melatonin as a therapeutic option for diabetic osteoporosis.

## Introduction

Osteoporosis results in fragile bones and is often characterized by deteriorated microarchitecture and reduced bone mass (Rachner et al., 2011; Langdahl, 2015). patients with osteoporosis, regardless of sex, those aged >50 years are particularly vulnerable to fragility fractures and bone loss (Eisman et al., 2012). Notably, in type 2 diabetes patients, the incidence of osteoporosis is significantly higher which is linked to associated metabolic changes in diabetes (Gong et al., 2016; Zhang et al., 2016). However, understanding of specific etiology and mechanism of diabetic-osteoporosis is still evolving.

Endoplasmic reticulum (ER) is intricately involved in the regulation of protein folding, protein translocation, and calcium (Ca_2_^+^) homeostasis (Gething and Sambrook, 1992; Ellgaard et al., 1999). Accumulation of misfolded proteins is a major hallmark of ER stress (Prins and Michalak, 2009; Zhou and Liu, 2014). To evade the growth of misfolded/unfolded proteins in the ER, eukaryotic cells elicit the unfolded protein response (UPR) (Ron, 2002; Rutkowski and Kaufman, 2004). The UPR comprises of three concurrent steps: inhibition of global protein synthesis to stop production of additional unfolded proteins, facilitate the refolding of unfolded proteins by stimulating the ER molecular chaperones, and activation of the ubiquitin-proteasome protein degradation pathways to remove the accumulated unfolded proteins. Failure of the UPR triggers ER stress-induced apoptosis.

The major regulators and proximal facilitators of the ER stress (ERS) response machinery are the protein kinase RNA-like endoplasmic reticulum kinase (PERK), eukaryotic initiation factor 2α (eIF2α), activating transcription factor 4 (ATF4), and C/EBP homologous protein (CHOP) (Harding et al., 1999; Ron, 2002; Zhang and Kaufman, 2008). During ER stress, upon phosphorylation and activation, PERK phosphorylates eIF2α at the Serine 51 (S51) position. This in turn leads to the inhibition of canonical translation initiation machinery (Shi et al., 1998; Harding et al., 1999). However, certain messenger RNA (mRNAs) harboring an upstream open reading frame (uORFs) in the 5′-untranslated region (UTR) can evade the global translation silencing. One such mRNA is ATF4 which is not inhibited by phosphorylated eIF2α (Harding et al., 2000; Vattem and Wek, 2004). Both CHOP and PERK mediates increase in pro-apoptotic BH3-only proteins, which in turn induces cytochrome-c release from mitochondria, initiating mitochondrial apoptotic pathway. CHOP also increases expression of GADD32 and ER oxidase 1 alpha which increases cellular calcium and reactive oxygen species (ROS) production, which also induces apoptotic death (Kojima et al., 2003; Marciniak et al., 2004; Li et al., 2009; Lin et al., 2009).

Melatonin (MLT; N-acetyl-5-mmRNAethoxytryptamine) is a neuroendocrine hormone secreted from the pineal gland that carries out a multitude of functions, including protection against oxidative stress, free-radical scavenging, apoptosis, and pro-inflammatory signaling (Hill et al., 2009; Galano et al., 2011; Mauriz et al., 2013; Mortezaee et al., 2019). Multiple studies suggest that melatonin can suppress ER stress to protect the brain, kidney, and myocardium against the ischemia/reperfusion injury (Hadj Ayed Tka et al., 2015; Yu et al., 2016; Lin et al., 2018). We have also earlier shown that a high concentration of melatonin induces osteoblast apoptosis while a low concentration promotes osteoblast proliferation (Liu et al., 2011; Liu et al., 2012; Meng et al., 2018; Tao and Zhu, 2018).

MLT exerts its action by binding to a type of G protein couple receptors, called the melatonin receptors, MT1 and MT2, on cell membranes. Binding of melatonin to MT1 and MT2 receptors induces downstream signaling pathways by intracellular mediators comprising of tubulin, calreticulin, calmodulin, and quinone reductase 2 (Pandi-Perumal et al., 2008). MT1 and MT2 knock-out mice indicated a critical role of MLT and these receptors in homeostasis of blood glucose (Muhlbauer et al., 2009). Furthermore, risk variants in *MTNR1B* (encoding MT2) has been reported in patients with type 2 diabetes (Mason et al., 2020). Indeed, treatment with MLT has been shown to restore metabolic dysfunction in elderly patients with hypertension (Shatilo et al., 2010), indicating a role of MLT/MT1/MT2 in metabolic regulation. Clinical trials of MLT supplementation in diabetes patients unequivocally indicated its role in regulating antioxidant enzymes (SOD, CAT, and GPx) and in increasing the overall antioxidant capacity (Rybka et al., 2016; Zare Javid et al., 2020).

The high concentrations of MLT in mitochondria provides further evidence of its role in anti-apoptotic and anti-oxidative stress (Reiter et al., 2016). Indeed, MLT has been shown to improve the glycemic state by upregulating GSH (Gurel-Gokmen et al., 2018). MLT levels in blood is lower in diabetic patients compared to healthy individuals (Cipolla-Neto et al., 2014; Reutrakul et al., 2018). One potential protective mechanism of MLT on blood glucose levels is mediated by MT1 and MT2 receptors-mediated regulation of GLUT4 expression and post-translational modification of the insulin substrate, in turn activating insulin signaling (Cipolla-Neto et al., 2014). We have recently demonstrated that treatment of the human fetal osteoblastic cell line hFOB 1.19 induces ERS and autophagy at an early stage which promotes survival, while at later stages there might be interaction between ERS and autophagy, in turn inducing apoptosis (Cui et al., 2020). However, in a high glucose environment, whether melatonin would affect ERS in osteoblasts is not known. Hence, using the osteoblastic cell line MC3T3-E1, we investigated the role and effect of melatonin in high glucose-induced ER stress.

## Materials and Methods

### Reagents and Cell Culture

Mouse osteoblastic MC3T3-E1 cells were obtained from the Shanghai Cell Bank, Chinese Academy of Sciences. These were cultured in α-MEM (Thermo Fisher Scientific, Carlsbad, CA, United States) medium with 10% fetal bovine serum (Thermo Fisher Scientific). All antibodies were purchased from Abcam (Cambridge, MA, United States). For T2DM-mimic culture conditions, cells were stimulated by the addition of 4.5 g/L glucose in the culture medium (Thermo Fisher Scientific). The cells were maintained in a humidified incubator at 37 °C and 5% CO_2_. As indicated, the MC3T3-E1 cells were either treated with MLT (100 nM; Sigma-Aldrich) or vehicle (0.2% DMSO). The dose was choosen as 100 nM as we and others have earlier shown that a dose of 10 nm—100 µM is optimal to study the effect of MLT (Liu et al., 2011; Quan et al., 2015; Xiong et al., 2015). Where indicated, cells were pre-treated for 30 min at 37 °C with 10 µM Luzindole (Sigma-Aldrich) before treatment with 100 nM melatonin. Dose of Lizindole was chosen based on previous study (Quan et al., 2015).

### Establishing MC3T3-E1 Cells Stably Expressing the S51A eIF2α

MC3T3-E1 cells were transduced with pGIPZ shRNA lentivirus to target the 3′-UTR of Eif2a using polybrene (Horizon Discovery, Cambridge, United Kingdom). The cells were selected using Puromycin (3 μg/ml) for 2 weeks and knockdown was verified by western blotting. The cDNA of mouse Eif2a was amplified by PCR and the amplicon was sub-cloned into the p3XFLAG-CMV-7.1 expression vector (Sigma-Aldrich). *Eif2a* S51A was generated by site-directed mutagenesis. The MC3T3-E1 cells, in which endogenous *Eif2a* was stably knocked down, were then transfected with the linearized p3X-FLAG-CMV-7.1-*Eif2a*_S51A plasmid and selection was carried out with G418 (100 μg/ml) and Puromycin for 6 weeks.

### Cell Viability Assay

Cell viability assays were carried out using the Cell Counting Kit-8 (CCK-8; Dojindo, Kumamoto, Japan) as per the manufacturer’s protocol. The viability was determined after 24 h of the indicated treatment conditions.

### Apoptosis Assay

Cell apoptosis was assessed using the Annexin V-FITC/PI method. The MC3T3-E1 cells, in the logarithmic growth phase, were seeded into a 100 mm dish. After 24 h of growth in DMEM/F12 medium containing 10% FBS, the cells were treated with melatonin for another 24 h. After this, the cells were harvested by centrifugation and washed twice with pre-cooled PBS. Then, these were stained with Annexin V and PI, and the flow cytometry (FACSCalibur, Becton-Dickinson, USA) was performed. The Annexin-V^+^/PI^−^, Annexin-V^+^/PI^+^, and Annexin-V^−^/PI^+^ cells were considered as the early, late, and necrotic apoptotic cells respectively.

### Detection of Intracellular Calcium Concentration

2 × 10^5^ MC3T3-E1 cells/well were seeded into 6-well plates. These were cultured in high glucose media with or without melatonin for 48 h. Post-incubation, the cells were trypsinized and stained with 200 μL of 5 μmol/L Fluo-4/AM (Beyotime, Shanghai, China) for 30 min at 37 °C. Finally, the cells were washed thrice with PBS, and the quantification was performed by excitation at 490 nm and emission at 525 nm respectively. Alternatively, calcium flux was determined by imaging using Fluo-4 Calcium Imaging Kit (Thermo Fisher Scientific) according to the manufacturer’s protocol.

### Real-Time Quantitative Polymerase Chain Reaction (RT-qPCR)

The total RNA was isolated using the TRIzol reagent (Thermo Fisher Scientific, Carlsbad, CA, United States) and reverse transcribed using the PrimeScript RT Reagent Kit (Takara Biotechnology co., Ltd., Dalian, China) following the manufacturer’s instructions. Real-time PCR was performed using the SYBR Premix Takara Ex Taq on the ABI Prism 7900HT Fast System (Applied Biosystems, Life Technologies, Foster, CA, United States). Amplifications were carried out using the primers reported previously (Sharma et al., 2008). *Gapdh* was used as an internal control and relative expression was calculated using the 2^−ΔΔcq^ method (Livak and Schmittgen, 2001).

### Western Blotting

At the indicated time points, the MC3T3-E1 cells were lyzed in buffer containing 150 mM NaCl, 1% NP-40, and 0.1% SDS, supplemented with protease and phosphatase inhibitors. The lysates were centrifuged at 12,000 g for 15 min at 4 °C and the total protein content of the lysates was measured. Then, 50 μg of protein lysates were resolved by 10% SDS-PAGE and the proteins were transferred onto PVDF membranes. GAPDH was used to confirm equal protein loading across the different samples. The protein blots were blocked in 5% skimmed milk and incubated with primary antibodies as indicated (all antibodies were used at 1:2000 dilution). Subsequently, incubation with secondary antibodies was performed and the protein bands were illuminated using the ECL reagent. Quantification of the band intensities was carried out using the NIH ImageJ software.

### Statistical Analysis

All experiments were performed with at least three biological replicates, also each of them incorporating at least three technical replicates. Data are represented as mean ± standard error of the mean (SEM). The differences between the groups were determined using the one-way analysis of variance followed by the Student Newman-Keuls test. *p* < 0.05 indicates a statistically significant difference between the samples.

## Results

### Melatonin Reverses the High Glucose-Induced Inhibition of Cell Proliferation and Increase in Apoptosis in the MC3T3-E1 Cells

**FIGURE 1:**
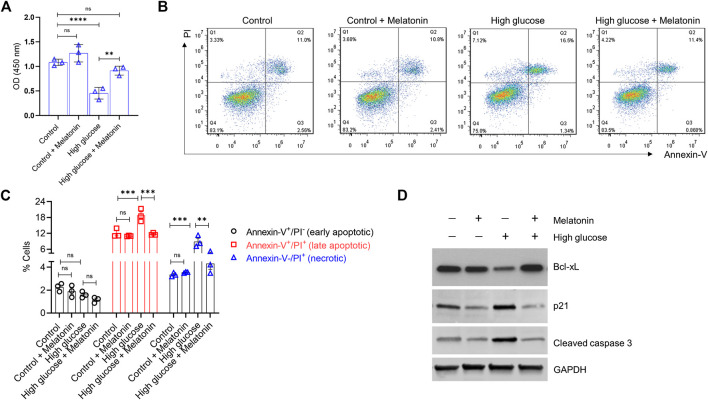
Melatonin reverses the high glucose-induced inhibition of cell proliferation and increase in apoptosis in the MC3T3-E1 cells **(A)** Cell proliferation was determined by the CCK8 assay for cells grown under normal (control) or high glucose conditions for 24 h ± melatonin (100 nM) **(B,C)** Apoptotic cell death was determined by Annexin-V/PI staining for cells grown under normal (control) or high glucose conditions for 24 h ± melatonin (100 nM) **(B)** The representative flow cytometry dot plots and **(C)** quantitative data of early apoptotic, late apoptotic, and necrotic cells are shown **(D)** Representative immunoblot of the anti-apoptotic protein Bcl-xL, pro-apoptotic proteins p21, and cleaved caspase three obtained from the MC3T3-E1 cell lysates grown under normal (control) or high glucose conditions for 24 h ± melatonin (100 nM) are shown. Results were obtained from three independent experiments. GAPDH was used as a loading control. For **(A,C)**, bars are mean ± SEM. **p < 0.01, ***p < 0.001, ****p < 0.0001, ns: not significant.

To determine the effect of high glucose on the proliferation in MC3T3-E1 cells, we first performed the CCK-8 assay. We found that high glucose significantly downregulated (2.38 ± 0.12 folds; *p* < 0.0001) cell proliferation compared to the control group of cells (Figure 1A). Treatment with melatonin rescued cell proliferation in the high glucose-treaed cells (2.01 ± 0.05 folds compared to untread cells, *p* < 0.01; Figure 1A). However, melatonin did not affect cell proliferation if added to the control group of cells growing under normal glucose conditions (Figure 1A; *p* > 0.05).

Next, we evaluated if high glucose-induced inhibition of cell proliferation would also be accompanied by induced cell apoptosis in the MC3T3-E1 cells. As expected, high glucose indeed showed a significant increase in both late apoptotic (1.57 ± 0.11 folds; *p* < 0.001) and necrotic (2.65 ± 0.12 folds; *p* < 0.001) cells (Figures 1B,C). Melatonin treatment significantly decreased high glucose-induced late apoptotic (1.61 ± 0.19 folds; *p* < 0.001) and necrotic (2.06 ± 0.31 folds; *p* < 0.01) cell death in the MC3T3-E1 cells. No effects of melatonin were observed in cells treated with normal glucose levels (Figures 1B,C). We did not observe a significant difference in the number of early apoptotic cells between the control and high glucose ± melatonin conditions (Figures 1B,C; *p* > 0.05). Moreover, when we tested these cells for the levels of apoptotic proteins using western blotting, we found that high glucose treatment led to a robust decrease in the level of the anti-apoptotic protein Bcl-xL but upregulated pro-apoptotic proteins p21 and cleaved caspase three in the MC3T3-E1 cells. However, melatonin treatment restored the expression of Bcl-xL and decreased the expression of p21 and cleaved caspase three respectively. (Figure 1D). Overall, these results indicate that melatonin treatment is capable of restoring the high glucose-induced effects on cell proliferation and apoptosis.

### Melatonin Alleviates High Glucose-Induced ER Stress

**FIGURE 2:**
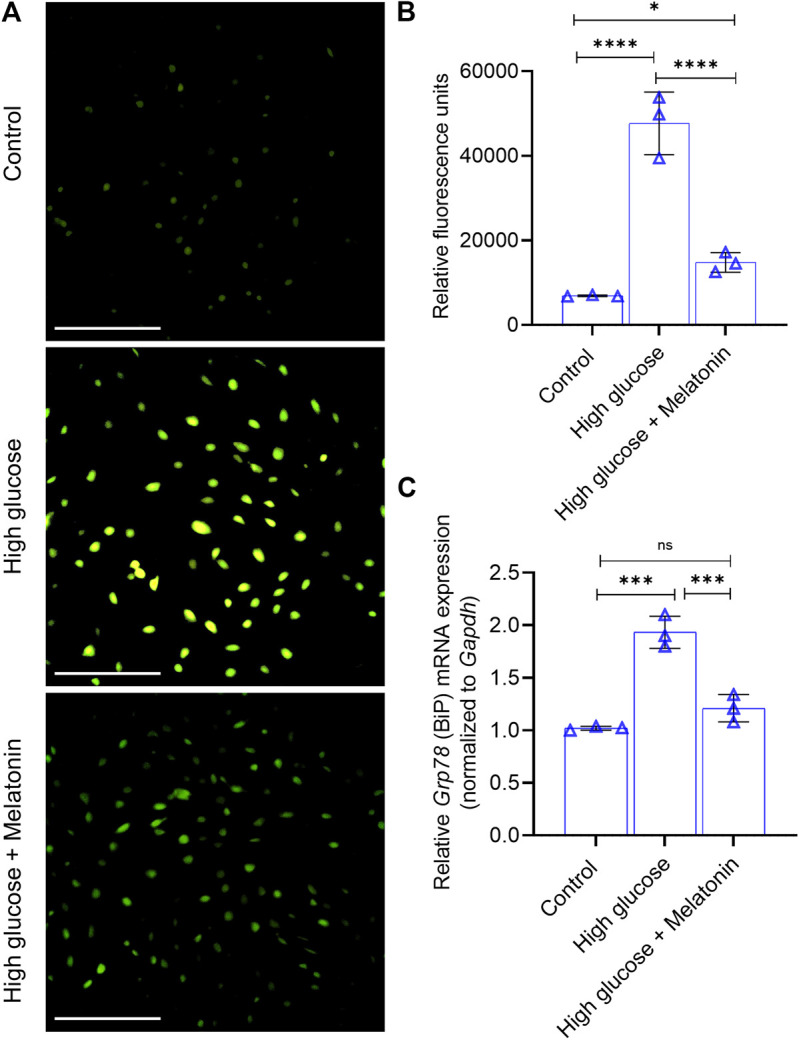
Melatonin alleviates high glucose-induced ER stress **(A,B)** Analysis of Ca_2_^+^ influx in the MC3T3-E1 cells grown under normal (control) and high glucose conditions for 48 h ± melatonin (100 nM) is shown using the **(A)** representative images obtained from Fluo-4 imaging (Scale bar: 50 µm) and **(B)** quantified spectrophotometrically using Fluo-4/AM **(C)** Relative mRNA expression of *Grp78* (which encodes BiP) is shown as indicated. The fold expression was calculated relative to the expression under control conditions. *Gapdh* was used as a normalization control. For **(B,C)**, bars are mean ± SEM. *p < 0.05, ***p < 0.001, ****p < 0.0001, ns, not significant.

Since Ca_2_^+^ influx is intricately related to osteoblast function, we next examined the changes in Ca_2_^+^ influx in the high glucose treated MC3T3-E1 cells. We found that high glucose treatment significantly increased (6.9 ± 1.2 folds; *p* < 0.0001) the Ca_2_^+^ influx in the MC3T3-E1 cells (Figures 2A,B), but this was markedly reversed (3.22 ± 0.81 folds; *p* < 0.0001) upon the addition of melatonin (Figures 2A,B). Altered Ca_2_^+^ homeostasis causes ERS which can be assessed by the expression of immunoglobulin heavy chain-binding protein BiP (also referred to as GRP78 or HSPA5). It is a member of the HSP70 protein family and is an established marker of protein folding and assembly in the ER during ER stress. We found that significantly upregulated (1.89 ± 0.23 folds; *p* < 0.001) BiP mRNA expression in the high glucose treated cells was markedly downregulated (1.61 ± 0.23 folds; *p* < 0.001) in the presence of melatonin (Figure 2C). Taken together, these results indicate that high glucose-induced ER stress can be alleviated by melatonin.

### Melatonin Protects Against High Glucose-Induced Cell Apoptosis via Inhibition of the PERK-eIF2α-ATF4-CHOP Pathway

ER, a major organelle with stress sensors, responds to cellular stressors. During ER stress, in UPR, activation of PERK leads to phosphorylation of eIF2α inhibiting canonical protein synthesis. However, the translation of ATF4 is not affected by such regulation due to an initiation site in its 5′-UTR sequences which is transcriptionally activated by phosphorylated ribosomal S6 kinase 2 (RSK2) and PKA. The activated ATF4 then upregulates C/EBP-homologous protein (CHOP) inducing apoptosis. PERK, ATF4, and CHOP are the proximal sensors of ER stress, whereas chaperone proteins like chaperone proteins HYOU1, HSPA5, DNAJC3, HSP90B1, CALR, and STC2 are downstream effectors (Sharma et al., 2008).

**FIGURE 3:**
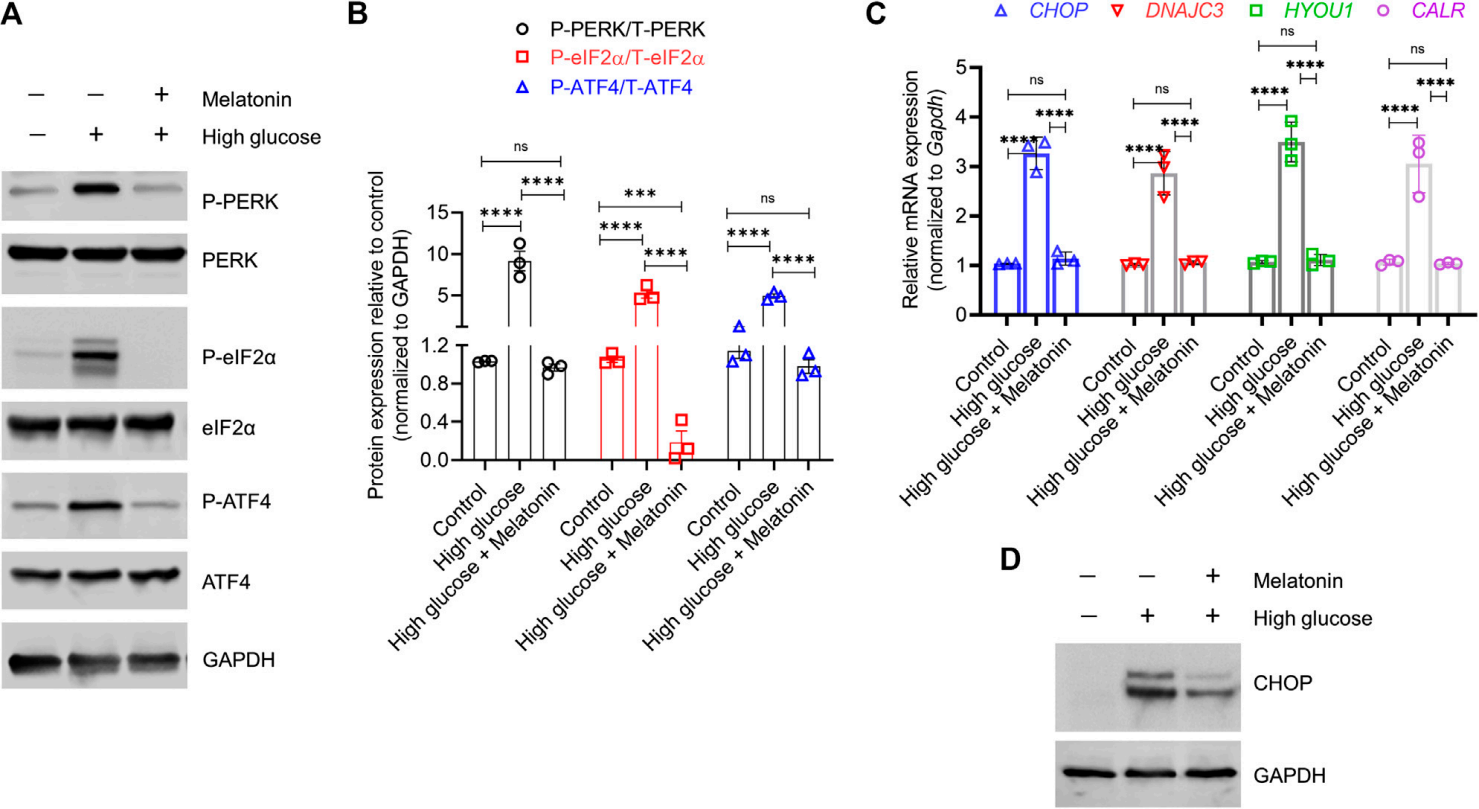
Melatonin reverses the activation of both proximal sensors and downstream effectors associated with high glucose-induced endoplasmic reticulum stress **(A,B)** Immunoblot analysis of phosphorylated and total PERK, eIF2α, and ATF4 proteins in the MC3T3-E1 cells grown under normal (control) and high glucose conditions for 48 h ± melatonin (100 nM) **(A)** The representative blots from three independent experiments and **(B)** the corresponding densitometry analysis are shown. GAPDH was used as a loading control **(C)** Relative mRNA expression of Chop, Dnajc3, Hyou1, and Calr in the MC3T3-E1 cells grown under normal (control) and high glucose conditions for 48 h ± melatonin (100 nM). The fold expression was calculated relative to the expression under control conditions. *Gapdh* was as used as a normalization control. **(D)** Representative immunoblot images of CHOP under the same conditions is shown. GAPDH was used as a loading control. These represent three independent experiments. For **(B,C)**, bars are mean ± SEM. ***p < 0.001, ****p < 0.0001, ns: not significant.

In high glucose treated MC3T3-E1 cells, we indeed observed a high level of P-PERK (8.84 ± 0.23 folds; *p* < 0.0001), P-eIF2α (4.91 ± 0.23 folds; *p* < 0.0001), and P-ATF4 (4.32 ± 0.34 folds; *p* < 0.0001) indicating induction of ERS (Figures 3A,B). However, the cellular levels of these proteins in the unphosphorylated state did not change. Melatonin treatment reversed the increase in the phosphorylated state of PERK (9.34 ± 1.23 folds; *p* < 0.0001), eIF2α (27.34 ± 0.13 folds; *p* < 0.0001), and ATF4 (5.03 ± 0.37 folds; *p* < 0.0001), without altering the unphosphorylated state (Figures 3A,B). Given that activated ATF4 upregulates transcription of downstream targets, we next examined the changes in the level of CHOP which is directly involved in ER stress-induced apoptosis. We found that high glucose treatment significantly increased CHOP mRNA exprssion (3.16 ± 0.21 folds; *p* < 0.0001); however, this was reversed by melatonin (2.86 ± 0.24 folds; *p* < 0.0001; Figure 3C). Similar observation was made for CHOP protein expression (Figure 3D). Similar changes in expression of the downstream effectors HYOU1, CALR, and DNAJC3 were observed (Figure 3C). Cumulatively, these results suggest a molecular model for melatonin mediated inhibition of the PERK-eIF2α-ATF4-CHOP signaling which otherwise triggers the ER stress-induced apoptosis.

**FIGURE 4:**
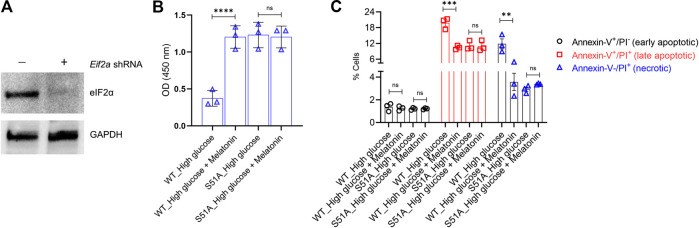
PERK-eIF2α-ATF4 axis is crucial for the protective action of melatonin **(A)** MC3T3-E1 cells were transduced with shRNA targeting the 3′-UTR of Eif2a. Successful knockdown was verified by immunoblotting. The representative blots from three independent experiments are shown. GAPDH was used as a loading control. **(B,C)** MC3T3-E1 cells in which endogenous *Eif2a* was stably knocked down were stably transfected with an *Eif2a*-S51A phospho-dead mutant expression constructed. MC3T3-E1 and MC3T3-E1/S51A cells were then grown under high glucose ± melatonin for 24 h **(B)** Cell proliferation was determined by the CCK8 assay and **(C)** apoptotic cell death was determined by Annexin-V/PI staining. For **(B,C)**, bars are mean ± SEM. **p < 0.01, ***p < 0.001, ****p < 0.0001, ns: not significant.

To test this hypothesis, we established MC3T3-E1 cells that could stably express the S51A mutant of *Eif2a* (encoding eIF2α). This was achieved by knocking down the endogenous wild-type *Eif2α* using the shRNA targeting the corresponding 3′-UTR (Figure 4A). These cells were denoted as MC3T3-E1/S51A and were grown under high glucose conditions. Surprisingly, high glucose treatment did neither decrease cell proliferation nor increased apoptosis in the MC3T3-E1/S51A cells (Figures 4B,C; *p* > 0.05 in each case). This phenomenon was completely reverse of the MC3T3-E1 cells expressing the WT *Eif2a* (3.31 ± 0.29 folds change in cell proliferation, *p* < 0.0001; and 1.89 ± 0.12 (*p* < 0.001) and 3.31 ± 0.12 (*p* < 0.01) folds change in late apoptotic and necrotic cells, respectively between control and high-glucose; Figures 4B,C).

**FIGURE 5:**
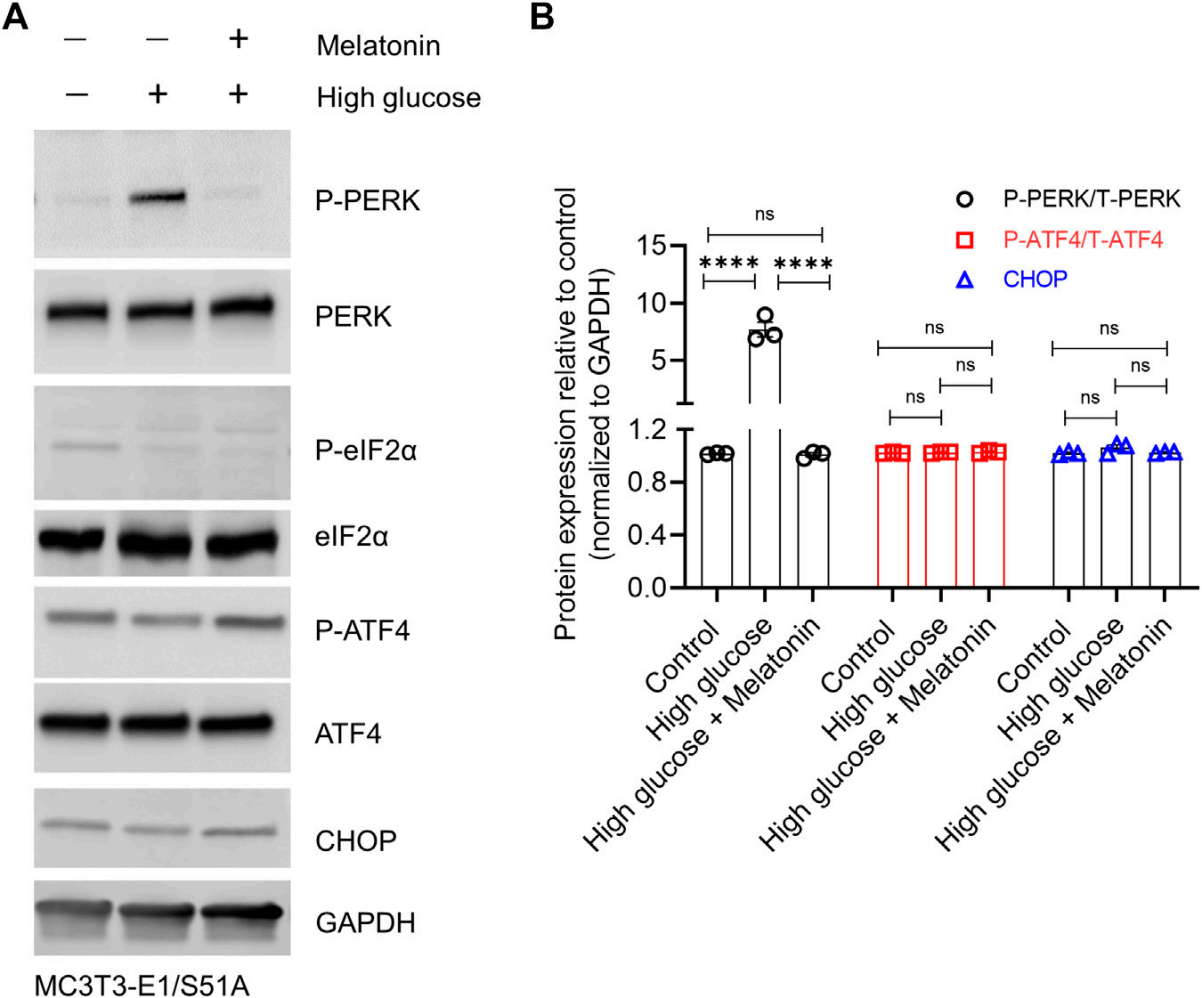
High glucose conditions could not induce ER stress in the MC3T3-E1 cells harboring the phospho-dead (S51A) mutant of Eif2a. MC3T3-E1/S51A cells were grown under normal or high glucose (±melatonin) concentration for 24 h. **(A)** Representative immunoblot images of phosphorylated and total PERK, eIF2α, ATF4, and CHOP are shown, and **(B)** densitometry analysis from three independent experiments are shown. GAPDH was used as a loading control. For **(B)**, bars are mean ± SEM. ****p < 0.0001, ns: not significant.

Besides, high glucose significantly increased the P-PERK protein level (7.56 ± 1.19 folds, *p* < 0.0001; Figures 5A,B), but failed to upregulate the downstream targets P-eIF2α, P-ATF4 and CHOP in the MC3T3-E1/S51A cells (Figures 5A,B; *p* > 0.05 in each case). This was again in contrast to the observations made in the MC3T3-E1 cells expressing the WT *Eif2α* (Figures 3A,B). Altogether, these results assert that high glucose-induced cell death in osteoblastic cell line MC3T3-E1 is via regulation of the PERK-eIF2α-ATF4-CHOP signaling pathway which can be alleviated by melatonin by directly impacting the regulation at PERK- eIF2α-ATF4-CHOP axis.

**FIGURE 6:**
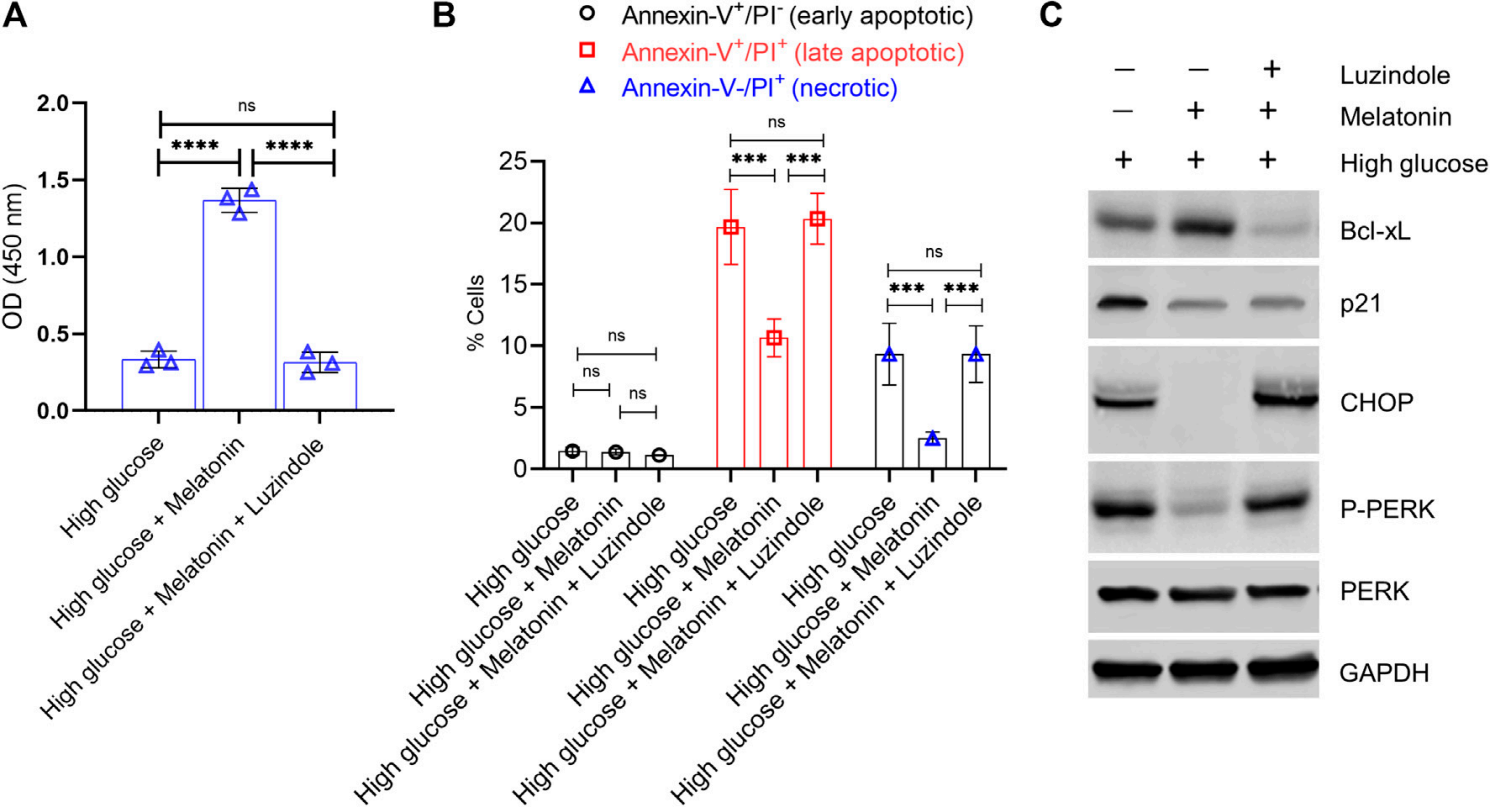
Protective action of melatonin against glucotoxicity in MC3T3-E1 cells is mediated via the high affinity melatonin receptors, MT1 and MT2. MC3T3-E1 cells grown under high glucose conditions were treated with melatonin (100 nM) for 24 h ± pre-treatment with Luzindole (10 µM for 30 min). **(A)** Cell proliferation was determined by the CCK8 assay. **(B)** Apoptotic cell death was determined by Annexin-V/PI staining. **(B)** Quantitative data of early apoptotic, late apoptotic, and necrotic cells are shown. **(C)** Representative immunoblot analysis of anti-apoptotic Bcl-xL, anti-apoptotic p21, and proximal ER stress markers P-PERK and CHOP. GAPDH was used as a loading control.

Given the inherent and critical role of the MT1 and MT2 receptors in mediating the action of melatonin and known correlation of these receptors to type 2 diabetes and regulation of blood glucose (Muhlbauer et al., 2009; Mason et al., 2020), we next determined if the protective functions observed in our experiments was being mediated by these receptors. We took advantage of a small molecule inhibitor of melatonin receptor, Luzindole, which has 25-fold greater affinity for the MT2 receptor compared to the MT1 receptor (Quan et al., 2015). Pre-treatment of MC3T3-E1 cells for 30 min with 10 µM Luzindole prevented melatonin-induced rescue of cell proliferation (4.36 folds less cell proliferation in melatonin + Luzindole compared to melatonin, *p* < 0.0001; Figure 6A). High glucose-induced apoptosis was not attenuated when cells were pre-treated with Luzindole (Figure 6B; *p* > 0.05). Immunoblot analysis showed that Lizindole pre-treatment prevented melatonin-mediated increase of anti-apoptotic protein Bcl-xL, decrease of pro-apoptotic p21, inhibition of ER stress induction (CHOP expression), as well as activation of PERK/eIF2α/ATF4 pathway (assessed by P-PERK expression) (Figure 6C). These results cumulatively indicate that the protective function of melatonin on osteoblast cells is mediated via the MT1/MT2 receptors.

## Discussion

The critical role of ER stress in osteoporosis pathogenesis has been recently elucidated (Li et al., 2017). Hence, it is essential to identify candidates that can reduce ER stress in osteoblasts. The results of this study show that melatonin could be a potential candidate for such a function. Our finding indicates that melatonin affects the regulation at PERK–eIF2α–ATF4-CHOP signaling axis and inhibits ER stress-induced apoptosis in osteoblasts. This action of melatonin was mediated via the high affinity MT1 and MT2 receptors. One limitation of our study was that we used only one dose of melatonin for all our experiments based on previous work by us and others (Liu et al., 2011; Quan et al., 2015). Dose and duration based response studies (of both melatonin and glucose concentrations) are required to clearly ascertain the kinetics of our observations. In addition, durability of response need to be ascertained by withdrawing melatonin after a fixed duration.

It has been previously reported that ER stress induces apoptosis in osteoblasts leading to osteoporosis (Joanna, 2018; Kim et al., 2018). In the MC3T3-E1 cells, high glucose treatment results in increased phosphorylation of eIF2α which in turn increases the transactivation of ATF4. Interestingly, in high glucose treated MC3T3-E1 cells, melatonin treatment modulates CHOP expression by attenuating the expression of P-PERK, P-eIF2α, and P-ATF4. This strongly indicates a melatonin-mediated protective role in osteoblasts against the high glucose-induced ER stress and apoptosis.

Oxidative stress and the associated generation of free radicals have been linked to ER stress in diabetic patients which ultimately leads to apoptotic cell death in osteoblasts (Burgos-Morón et al., 2019). The PERK-eIF2α mediates inhibition of canonical translation but activates the translation of ATF4 resulting in transcriptional activation of CHOP, a direct regulator of ER stress-induced apoptosis. The results from this study indicates that melatonin inhibits activation of ATF4; it remains to be determined if and how melatonin regulates oxidative stress under these conditions. Nuclear factor erythroid 2-related factor 2 (Nrf2), a senor of ocidative stress, is an important transcription factor that is phosphorylated by PERK in EIF2α-independent manner (Cullinan et al., 2003). Furthermore, it has been recently shown that Nrf2 signaling is critical, at least partially, for the biological and therapeutic effect of melatonin (Ahmadi and Ashrafizadeh, 2020). Hence, evaluating whether Nrf2 is involved in melatonin-mediated protective effect against glucotoxicity in osteoblasts is essential.

ATF4 activates transcription of CHOP which functioning as a transcription factor then downregulates the downstream anti-apoptotic factor Bcl-2 and upregulates the production of reactive oxygen species (ROS) (Hu et al., 2018). It has been reported that downregulating CHOP inhibits ER stress and apoptotic cell death both *in vitro* and *in vivo* (Hu et al., 2018). In this study, we observed that high glucose treatment upregulated CHOP leading to apoptosis in the MC3T3-E1 cells. However, melatonin treatment negatively affected the cellular CHOP levels and thereby reduced apoptosis along with the restoration of cell proliferation. Based on this, we propose a molecular model for melatonin functioning in which melatonin indirectly restores the expression of anti-apoptotic protein Bcl-2 to restore cell proliferation and inhibit cell death. It has been shown that p21 also contributes to UPR-adaptive signaling at initial stages of ER stress, whereas CHOP, besides inducing apoptosis, downregulates expression of p21, favoring a transition from UPR-adaptive pathways to apoptotic pattern (Mihailidou et al., 2010; Mihailidou et al., 2015). This suppression of p21 by CHOP was shown to be p53-independent (Mihailidou et al., 2015). We however, observed an increase in both CHOP and p21 expression under high glucose conditions and downregulation following melatonin treatment. This contrasting results might be due to the cellular context, and or duration of treatment.

During ER stress, melatonin maintains a steady-state through a variety of mechanisms. It is plausible that in addition to its regulatory effect on the PERK-eIF2α-ATF4 signaling pathway, it might also function through other pathways or factors. In conclusion, we showed that glucotoxicity-induced cell death and concomitant elevation of ER stress caused by an increase in calcium flux and activation of the PERK- eIF2α-ATF4-CHOP axis in osteoblast can be inhibited by melatonin treatment.

## Data Availability Statement

The original contributions presented in the study are included in the article/Supplementary Material, further inquiries can be directed to the corresponding author.

## Author Contributions

R-YZ and YZ designed experiments; YM and Z-BT carried out experiments; SQ, Z-LG and LT analyzed experimental results. R-YZ wrote the manuscript; YZ approved the manuscript. All authors read and approved the final manuscript.

## Funding

The study was supported by National Natural Science Foundation of China (Grant no. 81472044).

## Conflict of Interest

The authors declare that the research was conducted in the absence of any commercial or financial relationships that could be construed as a potential conflict of interest.
